# Targeting T cell (oxidative) metabolism to improve immunity to viral infection in the context of obesity

**DOI:** 10.3389/fimmu.2022.1025495

**Published:** 2022-10-06

**Authors:** Elizabeth Greene, Nancie J. MacIver

**Affiliations:** ^1^ Department of Pediatrics, Duke University School of Medicine, Durham, NC, United States; ^2^ Department of Pediatrics, University of North Carolina at Chapel Hill, Chapel Hill, NC, United States; ^3^ Department of Nutrition, University of North Carolina at Chapel Hill, Chapel Hill, NC, United States

**Keywords:** obesity, influenza, coronavirus disease 2019 (COVID-19), T cells, metabolism, metformin

## Abstract

Disorders of systemic metabolism can influence immunity. Individuals with obesity are known to have increased inflammation, increased risk to select autoimmune diseases, impaired response to several infections, and impaired vaccine response. For example, over the last decade, it has become clear that individuals with obesity have increased risk of morbidity and mortality from influenza infection. Unsurprisingly, this finding is also observed in the current COVID-19 pandemic: individuals with obesity, particularly severe obesity, have increased risk of poor outcomes from SARS-CoV-2 infection, including increased rates of hospitalization, ICU admission, mechanical ventilation, and death. Several studies have now demonstrated a critical role for T cells in the context of obesity-associated immune dysfunction in response to viral infection, and one mechanism for this may be altered T cell metabolism. Indeed, recent studies have shown that activated T cells from obese mice have an altered metabolic profile characterized by increased glucose oxidation, both *in vitro* and *in vivo* following viral infection. For that reason, treatments that target abnormal immune cell metabolism in obesity may improve outcomes to viral infection. To that end, several recent studies have shown that use of the metabolic drug, metformin, can reverse abnormal T cell metabolism and restore T cell immunity, as well as survival, in response to viral infection. These findings will be discussed in detail here.

## Introduction

Obesity is a significant public health concern due to both a high global prevalence and association with chronic disease. The prevalence of obesity has increased over the last few decades in the United States with the most recent CDC data showing that 42.4% of adults and 19.3% of children and adolescents have obesity (1). Moreover, obesity is the leading cause of preventable death and has become a global economic and health burden (2). Excess stores of adipose tissue in obesity lead to systemic low-grade inflammation which sets the stage for various pathological conditions, including type 2 diabetes mellitus, cardiovascular disease, nonalcoholic fatty liver disease, asthma, various types of cancer, neurodegenerative disease, and others (3). Although less well described, obesity is also associated with impaired immunity, characterized by increased risk of some autoimmune diseases (4–6), poor response to select infections (7, 8), and impaired vaccine response (8). As a specific example, obese individuals have a greater risk of morbidity and mortality from influenza (9–11). Unsurprisingly, this finding is also observed in the current COVID-19 pandemic; individuals with obesity, particularly severe obesity, have increased risk of poor outcomes from infection with SARS-CoV-2, including increased rates of hospitalization, ICU admission, mechanical ventilation, and death (12–17).

Several studies have now demonstrated a critical role for T cells in this setting, with primary and secondary T cell responses to influenza impaired in obese mice and humans (9, 18–20). One potential mechanism by which obesity may alter T cell response is through metabolic reprogramming. T cell function and metabolism are closely linked, and many studies over the last decade have clearly demonstrated that changes to T cell metabolism influence T cell response (21). More recent studies have described the effects of obesity on T cell metabolism and identified T cell metabolic reprogramming as a potential mechanism for impaired response to viral infection in obesity (22, 23). Understanding the mechanisms by which obesity leads to inflammation and impaired immune response to viral infection is necessary to identify targeted treatments to improve outcomes in this high-risk population.

## Obesity and influenza outcomes

In 2009, a novel influenza A (H1N1) virus caused pandemic influenza. A systemic review with meta-analysis performed during the pandemic showed that obesity significantly increased the risk of morbidity and mortality from H1N1 influenza infection (24). A global observational study published in 2011 presented H1N1 influenza data from approximately 70,000 patients requiring hospitalization, 9,700 patients admitted to intensive care units, and 2,500 deaths from 19 countries. This study reported high proportions of obesity among patients admitted to the ICU and among fatal H1N1 cases (25). These results supported the observation that obesity may be a risk factor for severe influenza disease. Further, a case-cohort study comparing rates of hospitalization and deaths from 2009 H1N1 influenza found that morbid obesity was associated with increased risk of hospitalization and death in adult patients (10). Notably, the authors found that individuals with morbid obesity, even in the absence of other chronic medical conditions, were at increased risk for severe outcomes due to 2009 H1N1 infection (10). Another study examined 534 adult patients hospitalized in California with H1N1 influenza virus and found that 51% of the hospitalized patients had a BMI ≥ 30 (11), which was 2.2 times higher than the prevalence of obesity in adults from California (23.2%) and 1.5 times higher than the prevalence of obesity in US adults (33% at the time). Furthermore, the percentage of hospitalized patients with BMI ≥ 40 was 2.5 times higher than the prevalence of BMI ≥ 40 in the US population, and the percentage of hospitalized patients with BMI ≥ 45 was 10 times higher than the prevalence of BMI ≥ 45 in the US population (11). Importantly, individuals with severe obesity (BMI ≥ 40) had increased likelihood of mortality from influenza infection following hospitalization from H1N1 influenza (11). In a separate meta-analysis describing associations of obesity and poor outcomes during the 2009 H1N1 pandemic, the authors found that obesity doubled the risk of ICU admission and/or death (26). As in previous studies, they concluded that these associations were stronger in severely obese patients (26).

Following these observations made during the 2009 H1N1 influenza pandemic, it became clear that patients with obesity also had worse outcomes following infection with seasonal influenza. One meta-analysis designed to study the association between obesity and influenza infection showed that patients with obesity had a significantly higher risk for influenza infection, as well as an increased risk for hospitalization following infection (27). Patients with BMI ≥ 40 had an even higher risk of hospitalization (27). Moreover, patients with obesity were at increased risk for severe outcomes related to influenza infection including increased likelihood of admission to the ICU, need for mechanical ventilation, and death (27). Again, morbid obesity was found to be a significant predictor for severe outcomes and death from influenza (27).

Although influenza vaccination provides the best method for protection against influenza infection, several studies have demonstrated that obesity impairs the response to vaccination (18, 20, 28). Indeed, obesity increases the risk for vaccine failure to influenza (18), hepatitis B (29) and tetanus (30). Adequate vaccine response depends on both B cell (antibody) and T cell (cellular) memory responses, and the mechanism for impaired vaccine response may be due to impaired T cell function, as T cells from obese adults vaccinated for influenza were less activated when stimulated with influenza virus compared to T cells from vaccinated healthy weight adults, and despite adequate serological response to vaccination (28). Moreover, individuals with obesity were found to be twice as likely to develop influenza infection than healthy weight controls, despite vaccination (28).

## Obesity and SARS-CoV-2 outcomes

Similar to influenza, multiple studies have demonstrated that COVID-19 patients with obesity have increased rates of hospitalization, ICU admission, mechanical ventilation, and death compared to COVID-19 patients with normal weight (12–17). This increased risk of severe outcomes correlates with BMI, with increasing risk of severity as BMI rises from the overweight category to severe obesity (13). Severe COVID-19 infections are associated with pro-inflammatory injury and cytokine storm, leading to complications such as COVID-19 pneumonia, acute respiratory distress syndrome, and multi-organ failure (31). One hypothesis is that obesity-associated inflammation increases the susceptibility to severe COVID-19 infection (32). Inflammatory cytokines such as IL-6 and TNF are already increased in patients who are overweight or obese (33). Several recent studies have shown that individuals with moderate to severe COVID-19 also have high levels of inflammatory cytokines, such as IL-6, CRP, TNF, and IL-17 (31, 34–36). A recent multi-omics study revealed that pro-inflammatory proteins associated with immune cell activation strongly contributed to more severe disease (37). IL-6 and MCP-1 were further identified in a separate study to strongly associate with fatality (38). In addition to acute infection, persistent heightened inflammation and T cell dysregulation occurs in individuals following COVID-19 infection (39–42). Thus, the already pro-inflammatory state observed in obesity could contribute to the overwhelming cytokine storm seen in severe COVID-19, although further studies are needed to clarify the role of inflammatory cytokines in individuals with obesity and SARS-CoV-2 infection.

## T cell dysfunction to viral infection in obesity

Data from a prospective study published in 2013 showed that individuals with obesity mounted a vigorous initial antibody response to influenza vaccination; however, they had a greater than four-fold decrease in vaccine titers at 12 months post vaccination compared to titers at 1 month (9). In comparison, less than 25% of healthy weight controls had a greater than four-fold decrease in vaccine titers at 12 months post vaccination compared to titers at 1 month. Moreover, vaccinated subjects with obesity had a lower percentage of cytotoxic CD8+ T cells, and these CD8+ cells produced lower amounts of IFN-γ and granzyme B, compared to normal weight subjects (9). This change in CD8+ T cell number and function is critical, as CD8+ T cells help slow the spread and reduce the severity of influenza infection by causing cytotoxicity of influenza infected cells. This study also showed that CD4+ T helper cells from subjects with obesity had defects in activation and function when challenged with pH1N1 *ex vivo* (9). Altogether, these findings suggest that abnormal T cell responses may explain, at least in part, the increased risk of morbidity and mortality from influenza infection in individuals with obesity (18). These human findings have been supported by work in a mouse model. Influenza-infected obese mice were found to accumulate fewer activated and functional memory T cells compared to lean mice, and did not maintain memory T cells over time, which resulted in increased mortality following a secondary influenza infection (20). Notably, this reduction in memory T cells includes resident memory T cells in the lung, which are critically important for protection against subsequent infections (22). These findings underscore the concern that current vaccine and vaccine approaches may be less effective in an increasingly obese population.

## Altered T cell metabolism in obesity

**Figure 1:**
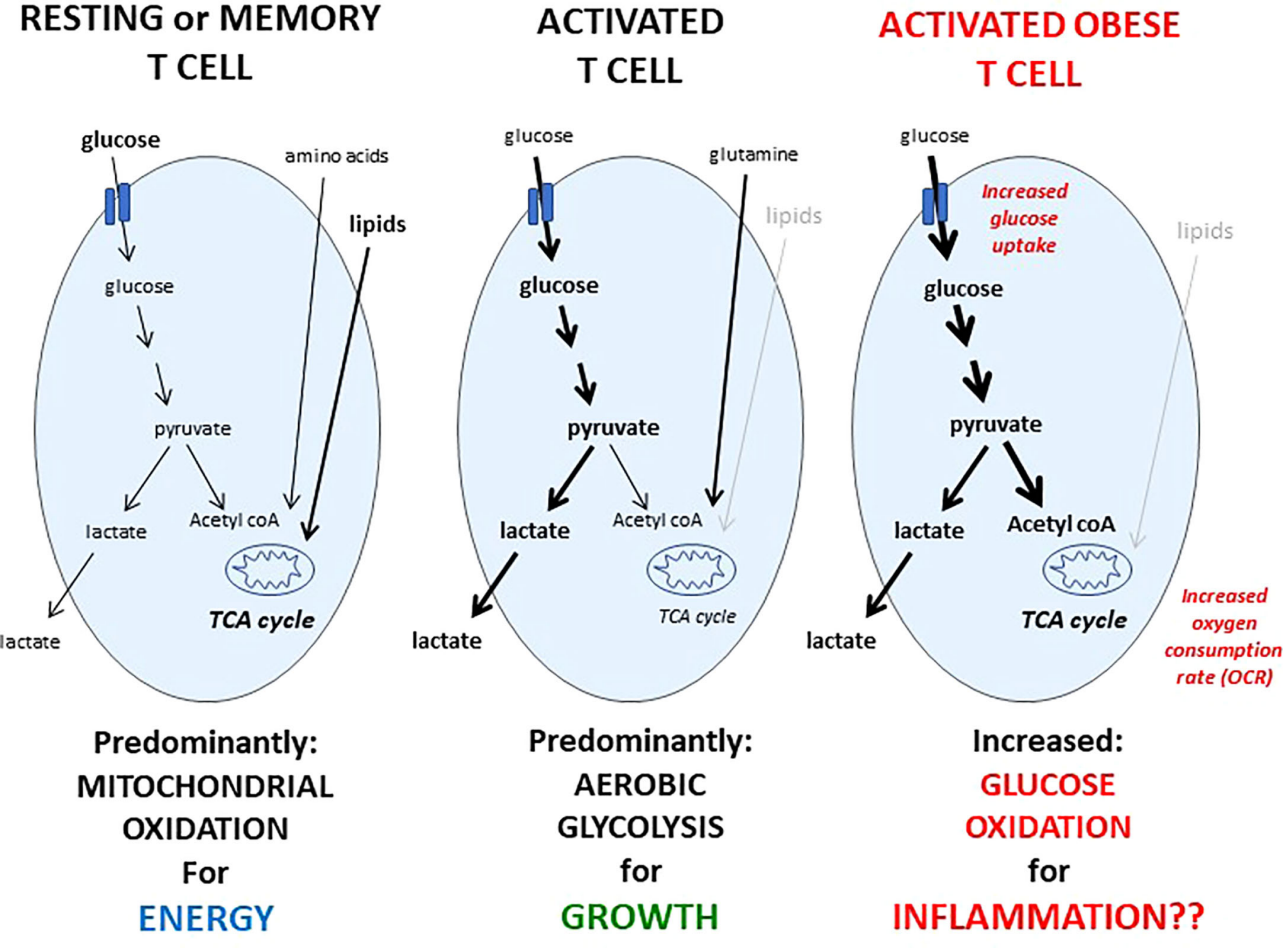
Metabolic phenotypes of resting, activated, or activated obese T cells. Resting T cells from lean humans or animals take up a mix of fuels: glucose, amino acids, and lipids, and predominantly metabolize these fuels in the mitochondria, which is an efficient way to generate ATP to fuel immune surveillance. Upon activation, the metabolic phenotype of a T cell changes, and glucose uptake and metabolism is significantly increased, although glutamine metabolism is also critical for T cell activation. In contrast to resting T cells, glucose taken up by activated T cells is converted into lactate in a metabolic program of aerobic glycolysis, which is a metabolic phenotype that generates critical biomass needed for growth, proliferation, and to produce critical factors needed for a successful immune response. Following activation, a portion of the T cell responders will become memory cells, and like resting T cells, memory T cells also rely on mitochondrial oxidation to fuel immune surveillance. Activated T cells from humans or mice with obesity demonstrate an increase in both glucose uptake and mitochondrial oxidation compared to activated T cells from lean animals, leading to a unique metabolic phenotype of glucose oxidation, which is not utilized by resting or memory T cells from lean animals, may explain mechanistically, at least in part, the T cell dysfunction seen in obesity, and may be targeted therapeutically.

A number of studies have demonstrated that T cell function and metabolism are closely linked, and that changes to T cell metabolism influence T cell activation, differentiation, and function (21). In recent studies performed in a mouse model of obesity, activated CD4+ T cells from diet induced obese mice had an altered metabolic profile characterized by increased glucose uptake, increased conversion of glucose to pyruvate, and increased mitochondrial oxidation (23). This represents a unique metabolic phenotype of glucose oxidation that is not utilized by T cells from lean animals and may mechanistically explain, at least in part, obesity-associated immune dysfunction **(** **Figure 1** **)**. Similar increases in mitochondrial oxidation were seen in CD4+ T cells isolated from obese mice following influenza reinfection (22). Interestingly, weight loss following a primary influenza infection in obese mice did not restore T cell memory response to influenza reinfection or reverse the obesity-induced changes in T cell mitochondrial metabolism (22). This suggests that obesity induces a unique abnormal T cell metabolic profile that cannot be reversed by weight loss.

Because weight loss was insufficient to reverse the obesity-associated abnormal T cell metabolism and function, other studies have attempted to use metabolic drugs to target T cell metabolism in obesity. One candidate was the commonly prescribed anti-diabetes drug, metformin. Metformin targets mitochondrial oxidation by disrupting electron transport in the mitochondria, presumably through inhibition of complex I of the electron transport chain (43). Inhibition of electron transport suppresses mitochondrial ATP production, and thereby activates the AMP-activated kinase (AMPK) (43), although metformin-induced activation of AMPK may not account for all actions of the drug (44, 45). Activated AMPK inhibits the activity of mammalian target of rapamycin (mTOR), a serine/threonine kinase which is a central regulator of growth, proliferation, and metabolism (46).

In recent studies, CD4+ T cells activated in the presence of metformin had decreased oxidative metabolism and changes in activation markers suggesting an improvement in memory potential, indicating a direct effect of metformin on T cell metabolism and function, and suggesting metformin might reverse T cell abnormalities in obese mice (23). Consistent with this, systemic treatment of obese mice with metformin was able to improve survival following an influenza challenge, compared to obese mice not treated with metformin or obese mice switched to low fat chow to induce weight loss (23). Moreover, CD4+ T cells isolated from influenza infected obese mice treated with metformin had normalization of T cell mitochondrial oxidation compared to untreated influenza infected obese mice (23), suggesting that metformin improved T cell immunity following infection, in part, through its ability to normalize T cell metabolism.

## SARS-CoV-2 and metformin

Metformin is a commonly prescribed drug for the treatment of type 2 diabetes mellitus and is also used off-label to treat obesity and obesity-associated disease. Metformin belongs to the biguanide class of antidiabetic drugs, has an excellent safety profile, is affordable, and is well tolerated by most patients. Studies have shown that metformin may also have other positive effects on health including anti-inflammatory and immune modulating effects. For example, metformin has been shown to lower inflammatory cytokines IL-6 and TNF while increasing the anti-inflammatory cytokine IL-10 (47). Because severe COVID-19 infection can lead to a pro-inflammatory cytokine storm, ultimately leading to acute respiratory distress syndrome and multi-organ failure (48), it is plausible to consider that the anti-inflammatory properties of metformin could be beneficial in this context.

In addition to its anti-inflammatory and immune modulating effects, metformin has also been shown to attenuate lung injury and reverse disease in a mouse model of lung fibrosis (49), suggesting that metformin might have an analogous benefit in improving acute respiratory disease in COVID-19. Metformin also improves glucose levels, reduces body weight, and reduces insulin resistance; all of which are important factors for improving immune responses and infectious outcomes (50). Additionally, metformin may confer protection against severe SARS-CoV-2 by extending the half-life of the ACE2 receptor. Although ACE2 is required for viral entry into cells, treatments to increase ACE2 have been shown to protect against severe cardiopulmonary complications of COVID-19 infections. In this setting, metformin works by activating AMP kinase (AMPK), which leads to phosphorylation of ACE2, enhancing its stability and increasing the expression of ACE2 protein (51).

Over the course of the COVID-19 pandemic, a number of studies have examined the effect of metformin on infection outcomes. Several retrospective studies have demonstrated reduction in mortality in patients with COVID-19 (47, 52–63). Some studies found a benefit in all patients taking metformin, but other studies only reported benefit in patients with obesity or type 2 diabetes (47, 52–57). Furthermore, several of these studies were systematic reviews and meta-analyses looking at large numbers of patients with COVID-19. One large meta-analysis looking at 5 studies and 6,937 patients showed by pooled analysis that metformin use is associated with reduction in the mortality rate from COVID-19 infections (60). The French nationwide CORONADO study observing 2,449 patients with diabetes hospitalized with COVID-19 infection showed a lower mortality rate on day 7 and 28 of illness among metformin users (56). Another large meta-analysis and systematic review looking at 28 studies with 2,910,462 individuals with COVID-19 infection showed a reduced risk of mortality and hospitalization in those taking metformin (61). A meta-analysis of 19 studies showed that metformin is associated with 34% lower COVID-19 mortality and 27% lower hospitalization rate (61). Another systematic review and meta-analysis including 9 studies and over 10,000 subjects across multiple countries found that metformin use was associated with lower mortality in hospitalized patients with COVID-19 (62). Finally, a meta-analysis including over 20,000 patients with diabetes found that metformin was associated with decreased mortality and severity in COVID-19 (54).

Two studies found a reduction in mortality among female patients only (64, 65). The authors postulated the reduction in mortality was due to metformin’s “sex-specific immunomodulatory actions”, in which the benefits of metformin are more significant in females than in males (64). Notably, metformin has been shown to reduce systemic TNF levels to greater degree in females than in males, which may explain, at least in part, the sex-specific benefit of treatment in female patients (64).

Although metformin is associated with an increased risk of developing lactic acidosis during illness, several studies looked at the question of whether or not to continue metformin treatment in patients hospitalized for COVID-19 (55, 66–68). One study found that the risk of lactic acidosis associated with metformin exposure was higher in patients with severe COVID-19 disease, patients with kidney impairment, and in patients taking more than 2 doses of metformin daily (66). Interestingly, several studies reported that in-hospital metformin therapy may be beneficial and reduce the risk of mortality in patients hospitalized with COVID-19 (55, 67, 68). It is therefore reasonable to consider continuing metformin therapy during hospitalization if kidney function is carefully monitored; however, further studies are needed before making definitive recommendations.

Other studies have shown improvement in COVID-19 outcomes in those taking metformin prior to hospital admission, including outcomes such as decreased rate of ICU admission (69) and decreased rate of hospitalization (70). Given the overwhelming number of studies showing an association with metformin and improved COVID-19 outcomes, the TOGETHER trial was conducted in Brazil in 2021. This placebo-controlled, randomized, clinical trial included 418 subjects, half randomized to taking metformin and half to placebo. There were no significant differences between the metformin and placebo groups on viral clearance through day 7 of illness and no difference in reducing emergency visits or reducing hospitalization for severe disease (71). This study suggests that acute administration of metformin may not be as beneficial as longer-term use of metformin.

## Discussion

Individuals with obesity are at increased risk of severe outcomes, including increased mortality, from both influenza and COVID-19. Because studies have shown that weight loss alone may be insufficient to reverse the immune dysfunction seen in obesity, there is a need to identify alternative therapeutic approaches to restore immune response to viral infections in the obese population. Although many immune cells are required for an effective anti-viral response, T cells are critical, and activated T cell function and metabolism are altered in obesity. The unique metabolic phenotype of glucose oxidation, which is not utilized by resting or memory T cells from lean animals but is seen in activated T cells from obese animals, may explain mechanistically, at least in part, the T cell dysfunction seen in obesity, and may be targeted therapeutically. Mouse studies have investigated the use of metformin to normalize T cell oxidative metabolism and restore immune response to viral infection in obese mice. Moreover, over the course of the last two years, several reports have shown that long-term use of metformin is associated with improved COVID-19 outcomes in humans. Although acute use of metformin does not appear to improve COVID-19 outcomes, long-term use of metformin in individuals with diabetes or obesity should be considered as potentially beneficial to both controlling systemic metabolism as well as improving immune responses to potentially life-threatening infections.

## Author contributions

EG and NM contributed substantially to the writing in this review article.

## Conflict of interest

The authors declare that the research was conducted in the absence of any commercial or financial relationships that could be construed as a potential conflict of interest.

## Publisher’s note

All claims expressed in this article are solely those of the authors and do not necessarily represent those of their affiliated organizations, or those of the publisher, the editors and the reviewers. Any product that may be evaluated in this article, or claim that may be made by its manufacturer, is not guaranteed or endorsed by the publisher.
